# Breast Cancer Diet “BCD”: A Review of Healthy Dietary Patterns to Prevent Breast Cancer Recurrence and Reduce Mortality

**DOI:** 10.3390/nu14030476

**Published:** 2022-01-21

**Authors:** Edda Cava, Paolo Marzullo, Deborah Farinelli, Alessandra Gennari, Chiara Saggia, Sergio Riso, Flavia Prodam

**Affiliations:** 1 Unit of Dietetic and Clinical Nutrition, University Hospital “Maggiore della Carità”, Corso Mazzini 18, 28100 Novara, Italy; deborah.farinelli@maggioreosp.novara.it (D.F.); sergio.riso@maggioreosp.novara.it (S.R.); 2 SCDU Endocrinology, Department of Translational Medicine, University of Eastern Piedmont, Via Solaroli 17, 28100 Novara, Italy; paolo.marzullo@med.uniupo.it (P.M.); flavia.prodam@med.uniupo.it (F.P.); 3 Laboratory of Metabolic Research, IRCCS Istituto Auxologico Italiano, 28824 Piancavallo, Italy; 4 Division of Oncology, University Hospital “Maggiore della Carità”, Department of Translational Medicine, University of Eastern Piedmont, Via Solaroli 17, 28100 Novara, Italy; alessandra.gennari@uniupo.it (A.G.); chiara.saggia@maggioreosp.novara.it (C.S.); 5 Department of Health Sciences, SCDU Endocrinology, Department of Translational Medicine, University of Piemonte Orientale, Via Solaroli 17, 28100 Novara, Italy

**Keywords:** breast cancer (BC), breast cancer survival, secondary prevention, healthy dietary patterns

## Abstract

Breast cancer (BC) represents the most common cancer in women, while overweight and obesity are the second preventable cause of cancer. Weight gain and fat accumulation are common after BC diagnosis; moreover, weight gain during the treatment decreases the survival rate and increases the risk of recurrence in breast cancer survivors (BCS). To reduce the risk of second primary cancer or BC recurrence, and all-cause mortality in BCS, multiple interventions have been investigated to obtain reduction in weight, BMI and/or waist circumference. The aim of this narrative review is to analyze evidence on BCS for their risk of recurrence or mortality related to increased weight or fat deposition, and the effects of interventions with healthy dietary patterns to achieve a proper weight and to reduce fat-related risk. The primary focus was on dietary patterns instead of single nutrients and supplements, as the purpose was to investigate on secondary prevention in women free from disease at the end of their cancer treatment. In addition, BC relation with insulin resistance, dietary carbohydrate, and glycemic index/glycemic load is discussed. In conclusion, obesity and overweight, low rates of physical activity, and hormone receptor-status are associated with poorer BC-treatment outcomes. To date, there is a lack of evidence to suggest which dietary pattern is the best approach for weight management in BCS. In the future, multimodal lifestyle interventions with dietary, physical activity and psychological support after BC diagnosis should be studied with the aim of reducing the risk of BC recurrence or mortality.

## 1. Introduction

Breast cancer (BC) represents the most common cancer in women, while overweight or obesity are the second preventable cause of cancer, related to 33% of postmenopausal breast cancers that could be prevented by lifestyle modification [1].

Excessive body fat deposition and weight gain promote a pro-oxidative microenvironment with chronic low-grade systemic inflammation, posing breast cancer survivors (BCS) at high risk of second primary cancer or BC recurrence, and all-cause mortality [2]. Healthy dietary patterns, weight loss intervention, and reduction of abdominal adiposity, instead, are related to a lower risk of BC recurrence and low-risk BC and all-cause mortality. 

The third expert report on “Diet, nutrition, physical activity and breast cancer” of the World Cancer Research Fund/American Institute for Cancer Research (WCRF/AICR) [dietandcancerreport.org] for preventing cancer generally recommends maintaining a healthy lifestyle by being physically active and eating a healthy diet for proper weight management [1]. 

Therefore, body weight management is crucial not only for primary prevention of cancer in general, but also for secondary cancer prevention in breast-specific sites. All women after a BC diagnosis undergo treatments for such a life-changing diagnosis and should be counselled to prevent excessive fat mass deposition, that is often a common event [3].

**Figure 1:**
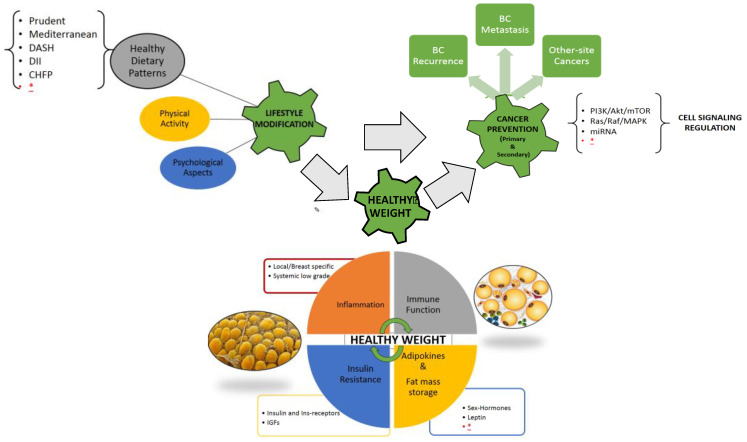
Visual Abstract, the complex interplay between lifestyle modification and healthy weight maintenance/achievement for primary and secondary cancer prevention; healthy dietary patterns together with physical activity and psychological support can affect multiple, and not yet fully known, physiological and pathological pathways related to the excess of adipose tissue (immune system, inflammation, adipokines and inflammatory cytokines, insulin resistance and metabolic homeostasis, cell cycle signaling). Legend: BC breast cancer, DASH dietary approach to stop hypertension, DII dietary inflammatory index, CHFP Chinese food pagoda, MiRNA micro-RNA, IGFs insulin-like growth factors, * further research needed.

The relation between overweight/obesity and cancer is still not fully clarified; the tumor environment could be metabolically stimulated by the excess of adipose tissue, with elevated levels of free fatty acids (FFA) and triglycerides, increased blood glucose, and insulin resistance. Increased production of adipokines, especially leptin, and inflammatory cytokines, such as tumor necrosis factor alpha (TNF-α), interleukin-6 (IL-6), interleukin-1 beta (IL-1β), TGF-β could exert local and systemic functions [4]. Breast adipose inflammation, elevated aromatase expression, dysregulated insulin signals, and particularly, increased levels of circulating leptin, raising in proportion to BMI and the total amount of body fat, have been identified as a possible key driver of this intricate network between excessive fat mass and breast cancer [5]. These factors interfering with cell signaling, namely the PI3K-AKT-mTOR pathway, which regulates cell-cycle progression, apoptosis, and protein synthesis can, at least in part, explain in women with high body fat mass the higher risk of cancer progression and metastasis, as well as other site-recurrence (Figure 1).

With this purpose, multiple interventions on breast cancer survivors (BCS) have been investigated especially in patients with overweight or obesity to obtain weight loss and reduce BMI and/or waist circumference as a proxy for proinflammatory fat accumulation. To date, there is still a lack of evidence to suggest which dietary strategy is the best among those available after BC diagnosis.

The aim of this review is to collect evidence on the risk of recurrence or mortality in BCS related to increased weight or fat deposition and the effects of interventions with healthy dietary patterns aiming to achieve a proper weight, improve waist circumference, or reduce obesity-related risk factors.

## 2. Methods

**Figure 2:**
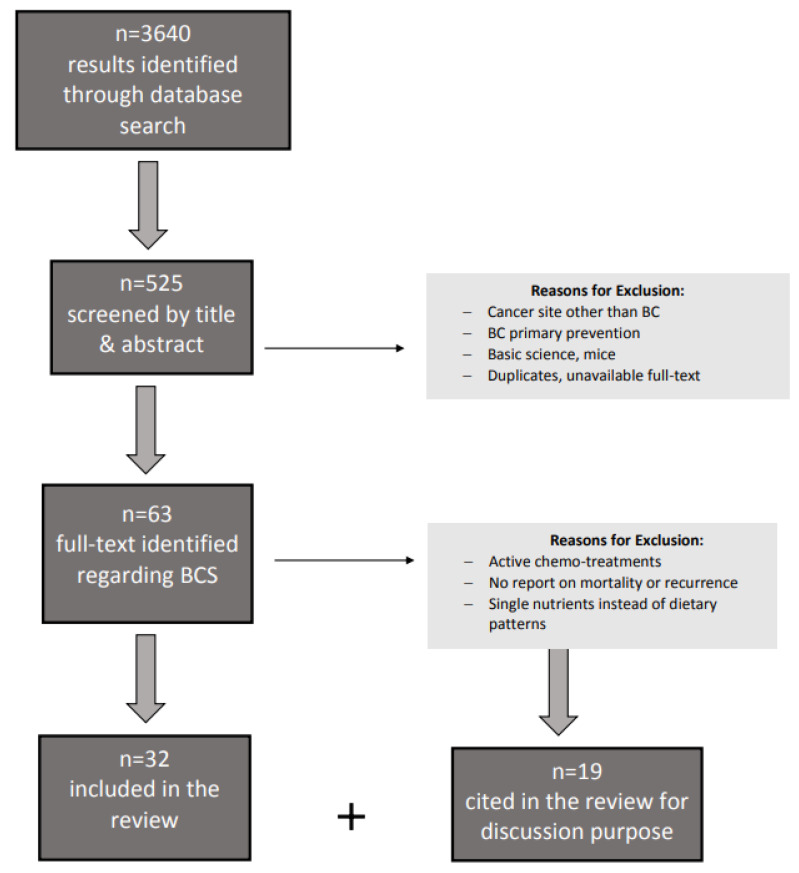
Flow diagram to describe paper identification and selection; Legend: BCS breast cancer survivors.

A search was conducted using keywords: “breast cancer” OR “breast cancer survivors” OR “breast cancer prevention” AND “nutritional therapy” OR “dietary therapy” OR “diet”, applying “humans” as a filter, and selecting articles published between 2015 and March 2021 on such databases as PubMed, Google Scholar, MEDLINE, EMBASE, and Scopus. Among 3640 results, 525 were included in the first selection after screening by title and abstract, 61 of which reported data on BCS. Among the latter, full texts of original studies and reviews were screened after exclusion of reports on “cancer” in general, thus only selecting those referring to site-specific “breast cancer”. In this narrative review, we assess the effect of dietary patterns to investigate secondary preventions in women free from BC or at the end of treatment. Studies were excluded if carried out on a BC patient on chemotherapy-active treatment, if reporting only single nutrients and/or supplement treatments, and if reporting only adherence to dietary recommendations/guidelines (dietary quality) or healthy patterns but not data on recurrence or mortality risk from BC. When included, “healthy patterns” were specifically described or specified (e.g., Mediterranean, Dietary Approach to stop Hypertension). Studies reporting outcomes other than recurrence or mortality risk (e.g., quality of life or psychological outcomes) were excluded. Eventually, duplicates, abstract without the full text available, reports on mice or basic science, and study protocols were excluded (Figure 2).

## 3. Results

Among 61 selected papers, only 32 address a specific dietary pattern implemented in BCS not undergoing chemotherapy or any active treatment other than hormonal or immune therapy, aimed at the prevention of recurrence. In Table 1, studies analyzing the relation between study outcomes and a specific food pattern or dietary index are listed (e.g., Mediterranean, DASH, DII) or a description of dietary characteristics of the intervention (e.g., fruit and vegetable intake). See also Appendix A.

## 4. The Role of Diet Quality in Breast Cancer Survivors

Women with BC show a 30% excess risk for second malignancies, even higher if considering contralateral breast cancers [25]. Weight gain during or after BC treatment increases the risk of recurrence and reduces the overall survival rate, while poorer BC survival has been associated with overweight and obesity [26]. While obesity and smoking are associated with cancer recurrence, physical activity represents the strongest driver to reduce BC recurrence and death. A report analyzing BCS stratified by age groups at diagnosis (<65 ≥ years), confirmed that all-cause mortality was significantly associated to BMI and physical activity, regardless if assessed pre- or post-diagnosis (in women ≥ 65 years, pre-diagnosis hazard ratio HR 1.27, 95% CI 1.14–1.41; post-diagnosis, HR 1.19, 95% confidence interval CI 1.04, 1.36); on the contrary, neither pre- nor post-diagnosis physical activity was associated with mortality [27]. 

In the European Prospective Investigation into Cancer and Nutrition (EPIC) cohort the risk for second malignancies after invasive BC was particularly elevated for colorectal cancer (OR 1.71), lymphoma (OR 1.80), melanoma (OR 2.12), endometrium (OR 2.18), and kidney cancers (OR 2.40), and positively associated with age at first cancer, BMI, and smoking status [25]. 

Another review investigating the best approach to recommend to overweight or obese BCS found that multimodal weight-loss interventions, including diet, exercise, and psychosocial support, achieved greater reduction in body weight, BMI, and waist circumference and improved overall quality of life more than dietary change alone [2]. Unfortunately, the analyzed reports were highly heterogeneous with a high risk of bias due to the study designs, with low-quality evidence.

Interestingly, a review has analyzed the association between dietary patterns and/or main food groups with mortality and cancer recurrence in survivors of common cancers, including BC, prior to or after cancer diagnosis. Their findings suggest that reducing the amount of body fat after diagnosis decreases the risk of breast cancer recurrence, and adherence to a high-quality diet, low-fat diet, or prudent diet after diagnosis is associated with a decreased risk of all-cause mortality in BCS. Conversely, adherence to a Western diet before and after diagnosis, is detrimental in terms of overall mortality risk and death from other causes among BCS [28]. Following a “prudent” dietary pattern is associated with a lower risk of overall death and death from causes other than breast cancer, while following a Western diet was a predictor of worse prognosis after breast cancer diagnosis [29]. 

Accordingly, a large prospective cohort study including 2295 postmenopausal women, the Women’s Health Initiative (WHI), with over 12 years of follow-up showed that adherence to a lower quality diet after BC diagnosis, as assessed through the Healthy Eating Index (HEI)-2010 score, increased the risk of death from BC (adjusted HR 1.66, 95% CI 1.09 to 2.52). Instead, a better quality diet (≥15% increase in HEI-2010 score) was non-significantly associated with a lower risk of death, irrespective of change in BMI [6]. A Chinese report collected five-year dietary information in BCS and found that healthy dietary patterns, namely Chinese Food Pagoda (CHFP) and the Dietary Approach to Stop Hypertension (DASH) had 25% to 34% lower risk of total mortality, and 36% to 40% lower risk of BC-specific events for a high score of adherence, while the Healthy Eating Index 2015 was not associated with reduced risks of both overall death and BC-specific recurrence or death among long-term BCS [7]. 

Another large-scale study enrolled 8927 women with stage I–III breast cancer in the Nurses’ Health Study (NHS; 1980–2010) and NHSII (1991–2011 follow-up) and assessed the associations of post-diagnostic fruit and vegetable consumption with BC-specific and all-cause mortality. The total fruit and vegetable and total vegetable consumption was related to lower all-cause [highest vs. lower quintile HR 0.82; 95% confidence interval (CI), 0.71–0.94; *p* for trend = 0.004, and HR 0.84; 95% CI, 0.72–0.97; *p* for trend = 0.001, respectively], but not related to BC-specific mortality. Total fruit consumption was not related to BC-specific or all-cause mortality. Greater intake of green leafy and cruciferous vegetables was associated with lower all-cause mortality, while higher fruit juice consumption, except for orange juice, which was associated with poorer BC-specific and all-cause survival [8]. 

In addition, higher adherence to healthy and anti-inflammatory dietary patterns, such as a Mediterranean diet, is associated to a higher quality of life [9]; however, an Italian retrospective cohort study with 1453 BCS did not show any association between the inflammatory potential of diets and women’s survival rates for both all-cause and BC-specific mortality after a median follow-up of 12.6 years [10]. 

To provide insight into the dietary inflammatory index (DII), a study assessed the risk for cancer recurrence and overall mortality after surgery for invasive BC finding significantly higher recurrence (HR 2.347, CI 1.17–4.71) and higher overall mortality (HR 3.049, CI 1.08–8.83) in patients with higher DII scores after adjusting for confounding factors, such as age (<50 years), premenopausal status, BMI (≥25 kg/m²), hormone receptor HR+, tumor size (>2 cm), and presence of lymph node metastasis [11]. A Korean report found a positive relation between DII and receptor positive BC for estrogen receptor (ER)+/progesterone receptor (PR)+ tumors regardless of menopausal status. The breast cancer odds ratio (OR) was higher in the highest DII tertile (OR = 3.68, 95% CI 2.34–5.80, *p* for trend <0.0001) than in the lowest tertile. Higher DII scores increased risk of (ER)+/(PR)+ breast cancer regardless of menopausal status (OR = 2.59 for premenopausal women; OR = 11.00 for postmenopausal women), but the risk was not increased for ER-/PR- status [12]. 

A low-fat dietary pattern (≤20% of total energy intake from fat) implemented on a long-term follow-up of 16.1 years was also able to reduce the incidence of deaths after BC in the intervention group compared to the usual diet [13]. 

A Danish study following up 1965 women for 7 years after BC diagnosis found that pre-diagnostic wholegrain intake of oatmeal/muesli was associated with lower all-cause mortality, while post-diagnostic intake of rye bread was associated with higher breast cancer specific mortality; moreover, a high intake of cheese was associated with a higher recurrence rate [14]. 

Another study observed the food intake trajectory for 2 years after BC diagnosis. Findings showed only a slight increase in fruit and vegetables and decreased alcohol intake, while total fat intake did not change post-diagnosis, with 45% of survivors maintaining a high-fat diet (fat > 40% of total calories) [30]. Dietary factors were not the only factors influencing diet after diagnosis, but sociodemographic, psychosocial, and other clinical factors, such as education, income, optimism, social support, physical well-being, and neuropathy symptoms also influenced dietary changes after BC diagnosis. The evidence supports the importance of proper psychological and nutrition counseling even after a BC diagnosis is made.

## 5. Breast Cancer Relation with Insulin Resistance, Dietary Carbohydrate, and Glycemic Index/Glycemic Load

Dietary patterns characterized by lower post-prandial glucose and insulin responses, those with low glycemic index (GI) and low glycemic load (GL), reflecting quality and quantity of carbohydrate intake, could help explain the reduction of mortality and recurrence risk in BCS. Mechanisms influencing BC development and recurrence include hyperglycemia, hyperinsulinemia, high insulin-like growth factor (IGF)-1, high circulating estrogen, inflammation, and impaired cellular differentiation/apoptosis [31]. Visceral adiposity and hyperinsulinemia have been pointed out as contributors that could promote and/or accelerate carcinogenesis, angiogenesis, and impair apoptosis. Metabolic syndrome and insulin resistance in obesity are characterized by an overexpression of insulin receptors, while insulin-related substrate (IRS), insulin, and IGFs are well-known influential factors for cell proliferation, differentiation, and regulation of the cell metabolism. Most breast cancer cells express IGF1 receptors, and IGF1 levels might be associated with an increased BC risk. Moreover, adipokines such as leptin, adiponectin, and other proinflammatory cytokines like tumor necrosis factor (TNF), interleukin (IL)-6, vascular endothelial growth factor (VEGF), and the hepatocyte growth factor are involved in tumor cell growth, apoptosis regulation, and neoangiogenesis. In addition, TNF-a, IL-6, and leptin can also increase aromatase levels and thus produce more estrogen, while insulin downregulates circulating levels of sex-hormone-binding globulin, thus elevating bioactive estradiol [32]. 

One meta-analysis of prospective cohort studies showed only a modest association between a dietary pattern with high GI or GL and the risk of breast cancer, even after adjustment for BMI, physical activity, and other lifestyle factors and for menopausal status and estrogen receptor status of the tumor [33]. Another meta-analysis found that GL and carbohydrate intake were positively associated with breast cancer among postmenopausal women with estrogen-negative tumors (relative risk RR for GL 1.28; 95% CI, 1.08–1.52; and summary RR for carbohydrates, 1.13; 95% CI, 1.02–1.25), independent of BMI [34]. 

A study on Mexican women concluded that total carbohydrate intake was associated with an increased risk of breast cancer among premenopausal women (OR 1.3, 95% CI 1.0–1.7; *p* trend = 0.03) with a positive association only among overweight women (OR 1.9, 95% CI 1.2–3.0; *p* trend = 0.01), but no association in women with BMI < 25 kg/m^2^ [35]. On the contrary, a similar analysis in more than 4000 postmenopausal women with obesity or diabetes at high CVD risk enrolled in the PREDIMED study, and yielded no significant associations of dietary GI and GL with an increased risk for invasive breast cancer in women [15]. 

## 6. Discussion

The association between dietary factors and breast cancer is weak, and etiologic mechanisms are still unclear. Preclinical and clinical data support the evidence that obesity may worsen the incidence, severity, and mortality of breast cancer. The dietary guidelines used as a reference is the WCRF [1], but evidence and guidelines for cancer prevention, are not specific to the BC population, and tend to overlap with DASH or Mediterranean dietary patterns (rich in vegetables, fruits, fish, whole grains, and unsaturated fats (especially coming from nuts and extra-virgin olive oil), with moderate red wine intake and limited intake of red meat and simple carbohydrates) [16,36], or the so-called “prudent” diet (high in fruits, vegetables, whole grains, and chicken) (Table 1). 

These healthy patterns rich in fruit and vegetables allow for a high intake of phytochemicals, such as polyphenols, which might reduce the risk of breast cancer incidence and recurrence [37]. In the “prudent” dietary patterns, major sources of polyphenols are olive oil, for phenolic acids (e.g., oleic acid, caffeic, oleuropein, hydroxytyrosol, oleocanthal) fruit and vegetables rich in flavonols (e.g., quercetin, kaempferol, myricetin, isorhamnetin), and isoflavones can be found in soy food. Isoflavones (genistein, daidzein, and glycitein) are considered natural estrogen receptor modulators, and defined as phytoestrogen, which can possibly protect against BC development, recurrence, and mortality. Unfortunately to date, further research in this field must be warranted, but more importantly, nutrients should be seen under a synergic point of view with different foods and different bioactive components interacting in a dietary pattern, and not only in separate analysis. The master regulator role of polyphenols is supported by recent evidence on the green-Mediterranean diet, an amplified version with green plant-based protein and polyphenols from Makai, green tea, and walnuts designed in the DIRECT PLUS randomized controlled trial. This modified version of the diet seems to increase the positive metabolic effects of the Mediterranean diet [38]. 

BC subtypes are mainly classified by hormonal receptor status: ER+ are BC with preponderance of tumor cells expressing estrogen receptors, and PR+ with cells expressing progesterone receptors, where HER2 cells carry human epidermal growth factor receptors [1]. Hormone receptor positive cancers are the most common subtypes of BC, while about 10% are found to be hormone receptor negative and show poorer outcomes, being difficult to treat and often of higher pathological grade. Moreover, BC is also classified by menopausal status at the time of diagnosis as pre- or post- menopausal. 

Being overweight or obese between the age of 18–30 years has shown an inverse association with the risk of developing pre-menopausal and post-menopausal BC (strong evidence), whereas in contrast, the risk of postmenopausal BC is increased for those with a body weight that is heavier than normal and those who gain weight in adulthood [1]. The mechanisms protecting against premenopausal BC women with greater body fatness are not yet well-known, but include implications of circulating levels of sexual hormones and binding globulin, as well as insulin-like growth factor 1 (IGF-1).

In postmenopausal women with obesity, the higher risk of estrogen receptor (ER)-positive BC is known, but similarly, increased adiposity in postmenopausal women with a normal body mass index also pose them at double risk of invasive BC (HR 2.21, 95% CI, 1.23–3.67). The group in the highest quartiles of trunk fat mass showed higher circulating levels of insulin, C-reactive protein, interleukin-6, leptin, and triglycerides, whereas levels of high-density lipoprotein cholesterol and sex hormone-binding globulin were lower [5]. Increased circulating levels of leptin with reduced levels of sex hormone-binding globulin and elevated expression of the estrogen synthesizing enzyme aromatase can increase the levels of free estradiol and possibly activate ERα stimulating cancer cell proliferation and survival in white adipose tissue accumulation and/or inflammation. Moreover, insulin resistance with dysregulated insulin signaling can activate the PI3K/Akt/mTOR and Ras/Raf/MAPK pathways [5,39,40,41,42].

Nevertheless, overweight and obesity are considered a risk factor for BC development, recurrence, and mortality, irrespective of hormone-receptor (HR) status. Women with obesity have a higher risk of developing triple-negative breast cancer (TNBC) anyway [42], a subtype of BC which, for growth and progression, is independent of estrogen, progesterone, and human epidermal growth factor 2 protein (HER2), and therefore is characterized by poorer prognosis with an increased risk of metastatic disease and lower survival rates. A review and meta-analysis also reported a 29% increased likelihood of death in overweight women with triple-negative breast cancer (TNBC) compared with patients of normal weight, showing both shorter disease-free survival (HR 1.26, 95% CI 1.09–1.46) and lower overall survival rates (HR 1.29, 95% CI 1.11–1.51) [42]. The connection between TNBC and adiposity could explain the pro-inflammatory microenvironment with a cytokines shift promoting growth and neoangiogenesis, stimulated also by increased levels of circulating free fatty acids [43].

A critical review suggests that nutritional therapy in BC patients should be based on the patients’ nutritional status, dietary habits, schedule, activities, and cultural preferences, to meet compliance and dietary adequacy for the improvement of overall health and prognosis [44]. Increased white adipose tissue in overweight and obesity, and increased levels of fat-specific cytokines, above all leptin, have been associated with proliferative signaling, inflammation, angiogenesis, that can sustain BC growth, tissue invasion, and metastasis [4]. High cancer recurrence risk among obese survivors can be driven by inflammatory cytokines, including C-reactive protein (CRP), Interleukins −3, −6, and −8 TNF-α [17]. Although diets with low inflammatory index showed non-BC-specific reduced cardiovascular mortality, compared to higher inflammatory scores, healthier dietary patterns can nonetheless improve overall survival rates in BC patients [45].

Circulating levels of IGFs and their binding proteins have been associated with BC risk, but evidence related to BC prognosis is still limited and inconsistent [46]. The IGF family is affected by fasting and the nutritional state; therefore, more research is needed to clarify the impact of these molecules in BC prognosis and how dietary patterns can affect it in the long term. The ER (+ or −) status of the women under study might also be another determinant in the relation between prognosis and circulating IGFs.

Although the presence of obesity increases the risk of cancer recurrence and death, its causative role is still not clear, and weight loss interventions deserve more research to clarify if benefits can be achieved through a combination of dietary intervention and aerobic exercise or by one intervention alone. A clinical study analyzing tumor markers (e.g., Ki67), gene expression on surgical specimens, blood cytokines, growth and metabolic factors found unclear benefits on tumor biology after pre-surgical caloric restriction in women undergoing a two-arm, single-blinded, randomized controlled and successful weight-loss trial [47]. The same study found some possible benefits of pre-surgical physical activity.

In order to shed some light on the link between BC and excess fat, in women with overweight or obesity, a study identified novel microRNAs associated to BMI and weight loss that could contribute to the development of cancer, identifying multiple pathways associated with cancer, and highlighting potential mechanisms explaining the link between BMI and increased cancer risk [48]. Among a pattern of 35 miRNAs, eight were associated with BMI, including miR-191-5p and miR-122-5p.

Another study worth mentioning is that which investigated the association between dietary acid load and inflammation as well as hyperglycemia in BCS, where a positive association was found in women with highest intakes of dietary acid load who showed a 30–33% increase in CRP and a 6–9% increase in HbA1c levels. This finding could provide a possible link explaining how dietary habits affect both systemic inflammation and hyperglycemia, a well-known risk factor for recurrence and reduction in both overall and disease-free survival in BCS [18]. The same group studied 3081 early-stage BCS enrolled in the Women’s Healthy Eating and Living study spanning 7.3 years of follow-up, and reported a higher BC recurrence if baseline HbA1c levels were ≥5.6% (HR 2.15, 95% CI 1.34–3.48 for potential renal acid load PRAL, *p*-value 0.01, and 2.31 CI 1.42–3.74 for net endogenous acid production NEAP, *p*-value 0.05) [18].

### Weight Gain and Weight Management during and after BC Diagnosis

Weight gain is common after BC diagnosis, and weight gain during treatment decreases the survival rate and increases the risk of recurrence [2,32]. Multiple mechanisms occur and contribute to weight gain and fat accumulation, including physical inactivity, decreased resting metabolic rate, overeating, hormonal changes, and chemotherapy. Obesity/overweight treatments should be provided in BC survivors. Large cohort studies with fairly long follow-up, like WHI [25], suggest the importance of diet quality even after BC diagnosis to decrease specific and overall risk of death. However, to date, evidence on pharmacological or surgical treatment for weight management in BCS needs to be integrated, as data are provided only for small sample sizes and require further investigation [49].

Weight management programs in BCS have been effective in reducing weight, body fat, waist, and hip circumference [2,19,20], and intervention based on Mediterranean Diet are also able to improve serum antioxidant capacity, cholesterol, and glycemic profile [21].

Although the purpose of this review was to focus on dietary patterns, WCRF underscores the importance of a healthy lifestyle comprised of behavioral components such as physical activity and smoking [1]. Accordingly, a study compared unhealthy vs. healthy behaviors and diet patterns in a 13-year follow-up, showing that unhealthy lifestyles, both dietary and behavioral, were associated with all-cause and breast-cancer-specific mortality (HR 1.4 and 1.2, respectively) [22]. 

Programs to induce weight loss in women after BC diagnosis have been proven to be efficient and feasible in improving anthropometric parameters, quality of life outcomes, and circulating biomarkers [19,20,50]. Significant improvement in insulin resistance biomarkers, measured through fasting insulin, area-under-the-curve insulin, and Homeostatic Model Assessment for Insulin Resistance (HOMA-IR) index can be achieved with just ≥5% weight loss [23]. 

Together with metabolic risk and insulin resistance improvements, leptin and adiponectin decrease were correlated with a decrease in BMI and increase in cardiorespiratory fitness [51]. 

Interestingly, a longitudinal study followed BCS in five survivorship periods since diagnosis (years ≤ 3; 3 to ≤6 years; 6 to ≤9, 9 to ≤12, and 12 to 15 years) and found that non-drinker and non-smoker BCS slightly increased after diagnosis, and only in the recent survivorship period were BCS significantly more physically active and they consumed more fruit, but were less likely to be classified in the healthy weight range (*p* < 0.01) and have increased total energy [52]. Therefore, long-term support for behavioral change and nutrition counseling to maintain healthy lifestyle choices and healthy weight in BCS may be helpful after BC diagnosis.

In the end, many studies have reported an improvement in multiple quality of life parameters in BCS following healthy dietary patterns. Discussion of these studies goes beyond the purpose of our review, but this evidence represents further important support for multimodal and nutrition counseling for BCS.

The main limit is that this is not a systematic review, therefore some bias of selection and interpretation might be present, as it does not strictly follow the PRISMA checklist statement. Another limit is that we included in the discussion studies enrolling non-triple negative breast cancer (TNBC) patients undergoing hormonal or immune therapy to prevent cancer recurrence, while TNBC patients are not always eligible for such protocols. Therefore, this may cause another interpretation bias, and may explain, at least in part, the worse outcomes in patients with TNBC. In the end, the small amount of reports included in the review does not allow to clearly select a specific diet to recommend in BCS and to respond to the main aim of the study. Nevertheless, this limit underlies the importance of the work itself as a trigger for more specific research on nutritional therapy for the prevention of recurrence in BCS, as well as in other cancer sites.

To date, studies on BCS are not enough to suggest which is the best diet to prevent recurrence of cancers of the breast and other sites after a cancer diagnosis. Nevertheless, Mediterranean [52] and other healthy and anti-inflammatory dietary patterns [11,27,29,36,37] have proven efficacy in primary prevention, and therefore might be taken as a reference to design future trials in BCS.

## 7. Conclusions

Obesity and overweight, lower rates of physical activity, and hormone receptor-status subtype are associated with poorer BC treatment outcomes. To date, there is a lack of evidence to suggest which dietary pattern is the best approach for weight management in BCS (Figure 1). In the future, multimodal lifestyle interventions with dietary, physical activity and psychological support after BC diagnosis are essential to meet the goal of reducing the risk of BC recurrence or mortality.

## Figures and Tables

**Table 1:** Trials analyzing outcomes of specific food patterns or dietary indexes in BCS.

Reference	Subjects (*n*)	Methods	Main Outcome	Dietary Index/Pattern
Sun et al. [6]	2295 postmenopausal women with invasive BC	HEI-2010 score assessed over a 12-year follow-up in the WHI (women’s health initiative) study	Decreased diet quality after BC diagnosis was associated with higher risk of death from breast cancer	Healthy Eating Index (HEI)-2010
Wang et al. [7]	3450 5-year BCS	Adherence scores to CHFP-2007, CHFP-2016, modified DASH, and HEI-2015	Higher adherence to CHFP and DASH dietary guidelines associated with reduced risk of overall death and BCr-specific recurrence or death among long-term BCS	Chinese Food Pagoda (CHFP) Healthy Eating Index (HEI) 2015 DASH diet
Farvid et al. [8]	8927 women with stage I–III BC identified during follow-up of the	Assessing post-diagnostic fruit and vegetable consumption in the Nurses’ Health Study (NHS; 1980–2010) and NHSII (1991–2011) with FFQ every four years	High fruit and vegetable consumption may be associated with better overall survival among BC patients, but not breast cancer-specific mortality. High fruit juice consumption may be associated with poorer prognosis.	
Porciello et al. [9]	309 women (stages I–III, mean age 52 ± 1 years, BMI 27 ± 7 kg/m^2^).	HRQoL was assessed with questionnaires measuring physical, mental, emotional and social factors: EQ-5D-3L, EORTC QLQ-C30, EORTC QLQ-BR23.	Higher adherence (PREDIMED score > 7) to the MedDiet in BCS is associated with better QoL (physical functioning, sleep, pain, well-being)	Mediterranean Diet (MD)
Zucchetto et al. [10]	1453 women	Retrospective cohort study FFQ over 12.6 years FU	No association between the inflammatory potential of diet and the survival of BC women.	Dietary Inflammatory Index (DII)
Jang et al. [11]	511 women undergoing BC-surgery	213 months follow-up	Anti-inflammatory diets may decrease the risk of cancer recurrence and overall mortality in BCS particularly in younger age, premenopausal status, obesity, HR+, tumor size > 2 cm, and lymph node metastasis.	Dietary Inflammatory Index (DII)
Lee et al. [12]	364 BC patients and 364 age-matched controls	FFQ	Higher DII scores were related to an increased risk of BC for ER+/ PR+ tumors regardless of menopausal status, but not for ER-/PR- status.	Dietary Inflammatory Index (DII)
Chlebowski et al. [13]	48,835 postmenopausal women, aged 50–79 years, with no prior breast cancer,	8.5 years FU in the WHI DM trial: Usual diet comparison group (60%) vs. dietary intervention group (40%) with reduced fat intake to 20% of energy and increase vegetable, fruit, and grain intake.	Low fat dietary pattern may reduce the risk of death for breast cancer in postmenopausal women.	Low-Fat Diet
Andersen et al. [14]	1965 women with BC	FFQ obtained up to three times, pre- and post-diagnostic, over a period of 18 years (median FU 7 years)	Pre-diagnostic intake of oatmeal/muesli was associated with lower all-cause mortality, and post-diagnostic intake of rye bread was associated with higher breast cancer specific mortality	
Castro-Quezada et al. [15]	4010 women, aged 60–80 years, at high risk for CVD disease, initially free from BC	137-item FFQ obtained in the PREvención con DIeta MEDiterránea (PREDIMED) study, International Tables of Glycemic Index (GI) and Glycemic Load (GL) values	No associations were found between baseline dietary GI/GL and invasive breast cancer incidence in postmenopausal women	
McCullough et al. [16]	4452 women with locally and regionally staged breast cancer	A nine-point score reflecting concordance with ACS dietary recommendations was calculated pre and post diagnosis	Diets consistent with ACS guidelines were not associated with breast cancer-specific mortality, but with other causes of mortality.	ACS recommendations for cancer prevention
Zheng et al. [17]	2150 postmenopausal women with invasive BC, aged 50–79 years	FFQ on average 1.5 years after diagnosis a median 13.3 years of follow-up	Consuming a more anti-inflammatory diet after breast cancer diagnosis may be a means for reducing risk of death from CVD	Dietary Inflammatory Index (DII)
Wu et al. [18]	3042 BCS	Cross-sectional study with dietary intake in the Women’s Healthy Eating and Living (WHEL) Study	Positive associations between dietary acid load and CRP and HbA1c in BCS, as strong risk factors for BC recurrence and comorbidities	Dietary acid load (DAL)
Finocchiaro et al. [19]	100 BCS	MD intervention with a 6 month follow-up	MD is effective in reducing BMI and waist circumference, and enhancing healthy lifestyle in BCS	Mediterranean Diet (MD)
Thompson et al. [20]	249 post-menopausal BCS	6-month non-randomized, controlled weight loss intervention with two dietary interventions, LFD and LCD	Loss of body weight and fat mass was effective irrespective of dietary approach on a structured program with monthly assessments	Low-fat (LFD) Low-carbohydrate diet (LCD)
Skouroliakou [21]	70 BCS randomized to MD or control group for 6 months	Anthropometric and biochemical parameters (vitamin C, vitamin A, a-tocopherol and CoQ10 levels, dietary intake and adherence to MD	MD ameliorate serum antioxidant capacity, body composition and glycemic profile of postmenopausal BCS	Mediterranean Diet (MD)
Parada et al. [22]	1808 women with invasive BC	Interviews to assess lifestyle and dietary patterns in the Carolina Breast Cancer Study Phases I/II, 13-year FU	The unhealthy (vs. healthy) behavior and diet pattern was associated with all-cause mortality and with BC-specific mortality	
Dittus et al. [23]	74 post-menopausal BCS, age ≤ 65 years	A 24-week Internet-based behavioral weight loss (BWL) intervention	Behaviorally based weight loss interventions can result in improvements in biomarkers in BCS who achieved ≥5% weight loss and demonstrated significant improvements in insulin resistance	
Toledo [24]	4282 women at high cardiovascular disease risk, aged 60–80 years	Randomized, single-blind, controlled trial with a low-fat diet (control) vs. 2 MD diet intervention 1:1:1 with 4.8 years FU	Beneficial effect of a Mediterranean diet supplemented with extra-virgin olive oil in the primary prevention of BC	Mediterranean Diet (MD)

Legend: Breast Cancer (BC); BC survivors (BCS); Follow-up (FU); Food Frequency Questionnaires (FFQ), American Cancer Society (ACS); World Cancer Research Fund/American Institute for Cancer Research (WCRF/AICR), Health Related Quality of Life (HRQoL), Quality of Life (QoL), EQ-5D-3L, EORTC QLQ-C30, EORTC QLQ-BR23 are HRQoL (see reference for related information).

## Appendix A. Dietary Index and Dietary Pattern Description

The Healthy Eating Index (HEI) measures the quality of diets following key recommendations of the Dietary Guidelines for Americans (for those aged 2 years and older). Developed in 1995, HEI has been periodically updated (HEI-2005, HEI-2010) with the latest version being HEI-2015. A high total score reflects the overall diet quality with a maximum of 100 points. (https://www.fns.usda.gov/healthy-eating-index-hei accessed on 26 November 2021).

The Dietary Inflammatory Index (DII) estimates the inflammatory potential of diets by completing a food frequency questionnaire. Proinflammatory diets are related to chronic inflammation, which is a risk for chronic diseases, including cancer, diabetes, and cardiovascular disease.

The Dietary Acid Load (DAL) represents the food potential to produce acid or base in the body—foods rich in protein (e.g., meat, cheese, eggs) increase the production of acid, whereas fruit and vegetables increase alkalis. It can be calculated by the Potential Renal Acid Load (PRAL) of food.

The Chinese Food Guide Pagoda (CFGP), proposed by the board of Chinese Nutrition Society, was first released to the public in 1989. It represents the Chinese Dietary Guidelines (CDGs) for the general Chinese population aged 2 years and above. Its latest update was in 2016.

The Dietary Approaches to Stop Hypertension (DASH diet) is a healthy-eating plan mainly developed to help, treat, or prevent hypertension and reduce the risk of cardiovascular disease. It is rich in fruits, vegetables, and lean proteins, and restricts red meat, salt, and added sugars and fat.

The Mediterranean Diet (MedDiet) was recognized by UNESCO as an intangible cultural heritage of humanities in 2010. This dietary pattern is characterized by an abundance of fruit, vegetables, legumes, cereals, and nuts, and the usage of olive oil is noteworthy, as well as the frequent consumption of fish, moderate consumption of dairy derivates, and low consumption of red meat and simple sugars (https://www.med-diet.eu/ accessed on 26 November 2021).

The term “Prudent Diet” has been in use since 1957 to describe a fat- and cholesterol-controlled diet. It is a generic term to identify healthy dietary patterns (including the MedDiet and DASH diet) characterized by a high intake of whole grains, fruits, vegetables, legumes, nuts, fish, and low-fat dairy products, with low intake of processed foods, red meats, products high in sugar, and fats.
